# Identification of Germline Mutations in Melanoma Patients with Early Onset, Double Primary Tumors, or Family Cancer History by NGS Analysis of 217 Genes

**DOI:** 10.3390/biomedicines8100404

**Published:** 2020-10-09

**Authors:** Lenka Stolarova, Sandra Jelinkova, Radka Storchova, Eva Machackova, Petra Zemankova, Michal Vocka, Ondrej Kodet, Jan Kral, Marta Cerna, Zuzana Volkova, Marketa Janatova, Jana Soukupova, Viktor Stranecky, Pavel Dundr, Lenka Foretova, Libor Macurek, Petra Kleiblova, Zdenek Kleibl

**Affiliations:** 1 Institute of Biochemistry and Experimental Oncology, First Faculty of Medicine, Charles University, 128 53 Prague, Czech Republic; lenka.stolarova@lf1.cuni.cz (L.S.); sandra.jelinkova@lf1.cuni.cz (S.J.); petra.zemankova@lf1.cuni.cz (P.Z.); jan.kral@lf1.cuni.cz (J.K.); marta.cerna@lf1.cuni.cz (M.C.); zuzana.klusonova@lf1.cuni.cz (Z.V.); marketa.janatova@lf1.cuni.cz (M.J.); jana.soukupova@lf1.cuni.cz (J.S.); 2 Laboratory of Cancer Cell Biology, Institute of Molecular Genetics of the Czech Academy of Sciences, 142 20 Prague, Czech Republic; radka.storchova@img.cas.cz (R.S.); libor.macurek@img.cas.cz (L.M.); 3 Department of Cancer Epidemiology and Genetics, Masaryk Memorial Cancer Institute, 656 53 Brno, Czech Republic; emachack@mou.cz (E.M.); foretova@mou.cz (L.F.); 4 Department of Oncology, First Faculty of Medicine, Charles University and General University Hospital in Prague, 128 08 Prague, Czech Republic; michal.vocka@vfn.cz; 5 Department of Dermatology and Venereology, First Faculty of Medicine, Charles University and General University Hospital in Prague, 128 08 Prague, Czech Republic; ondrej.kodet@vfn.cz; 6 Institute of Anatomy, First Faculty of Medicine, Charles University, 128 00 Prague, Czech Republic; 7 BIOCEV, First Faculty of Medicine, Charles University, 252 50 Vestec, Czech Republic; 8 Research Unit for Rare Diseases, Department of Paediatrics and Inherited Metabolic Disorders, First Faculty of Medicine, Charles University and General University Hospital in Prague, 121 00 Prague, Czech Republic; Viktor.Stranecky@lf1.cuni.cz; 9 Department of Pathology, First Faculty of Medicine, Charles University and General University Hospital in Prague, 128 00 Prague, Czech Republic; pavel.dundr@vfn.cz; 10 Institute of Biology and Medical Genetics, First Faculty of Medicine, Charles University and General University Hospital in Prague, 128 00 Prague, Czech Republic; petra.kleiblova@lf1.cuni.cz

**Keywords:** melanoma, familial melanoma, hereditary cancer predisposition, germline mutations, panel sequencing, NGS

## Abstract

Cutaneous melanoma is the deadliest skin malignity with a rising prevalence worldwide. Patients carrying germline mutations in melanoma-susceptibility genes face an increased risk of melanoma and other cancers. To assess the spectrum of germline variants, we analyzed 264 Czech melanoma patients indicated for testing due to early melanoma (at <25 years) or the presence of multiple primary melanoma/melanoma and other cancer in their personal and/or family history. All patients were analyzed by panel next-generation sequencing targeting 217 genes in four groups: high-to-moderate melanoma risk genes, low melanoma risk genes, cancer syndrome genes, and other genes with an uncertain melanoma risk. Population frequencies were assessed in 1479 population-matched controls. Selected *POT1* and *CHEK2* variants were characterized by functional assays. Mutations in clinically relevant genes were significantly more frequent in melanoma patients than in controls (31/264; 11.7% vs. 58/1479; 3.9%; *p* = 2.0 × 10^−6^). A total of 9 patients (3.4%) carried mutations in high-to-moderate melanoma risk genes (*CDKN2A*, *POT1*, *ACD*) and 22 (8.3%) patients in other cancer syndrome genes (*NBN*, *BRCA1/2*, *CHEK2*, *ATM*, *WRN*, *RB1*). Mutations in high-to-moderate melanoma risk genes (OR = 52.2; 95%CI 6.6–413.1; *p* = 3.2 × 10^−7^) and in other cancer syndrome genes (OR = 2.3; 95%CI 1.4–3.8; *p* = 0.003) were significantly associated with melanoma risk. We found an increased potential to carry these mutations (OR = 2.9; 95%CI 1.2–6.8) in patients with double primary melanoma, melanoma and other primary cancer, but not in patients with early age at onset. The analysis revealed affected genes in Czech melanoma patients and identified individuals who may benefit from genetic testing and future surveillance management of mutation carriers.

## 1. Introduction

With 287,723 newly diagnosed cases and 60,712 fatalities in 2018, cutaneous melanoma remains the deadliest skin malignity globally. The highest standardized melanoma incidence occurs in Australia and New Zealand; however, European and US patients account for more than 75% of new melanoma cases annually [1]. The GLOBOCAN cancer registry ranks the Czech Republic as 18th among 185 countries in the world in terms of age-standardized melanoma incidence rates (between the USA and Canada) [2].

The risk of melanoma is largely modified by factors influencing individual sensitivity to UV radiation and sunlight exposure, and sunburns during childhood in particular are a major behavioral risk factor [3]. Other individual host factors include the amount, type, and arrangement of cutaneous melanin, the presence of multiple atypical moles (the most frequent precancerous melanoma lesions), and a family history of melanoma [4].

The hereditary component of melanoma development has been assessed in a large prospective study of twins from Nordic countries revealing melanoma heritability with a familial cancer risk of 19.6% and 6.1% for monozygotic and dizygotic twins, respectively, compared with 1.2% for the overall population [5]. The proportion of familial melanoma cases is approximately 5–10%; however, pathogenic germline mutation carriers have been identified in only a minority of the analyzed familial melanoma cases [6]. 

The major melanoma-susceptibility gene is *CDKN2A*, coding for two alternatively transcribed mRNAs translated into the cyclin-dependent kinase inhibitor p16^INK4^ and the tumor suppressor p14^ARF^ participating in p53 activation, respectively [7]. Germline *CDKN2A* mutations have been found in about 20–40% of melanoma-prone families (with ≥3 melanoma cases), but in only 0.2–3% of non-familial melanoma cases [8,9]. Other high-risk but extremely rare germline mutations affect cyclin-dependent kinase 4 (*CDK4*) and BRCA1-associated protein 1 (*BAP1*) genes [10,11]. Germline *CDK4* mutations cluster in exon 2, coding for a domain interacting with p16^INK4^ [12]. The BAP1 protein codes for deubiquitinase, counteracting BRCA1-BARD1 ubiquitin ligase activity [13]. Hereditary *BAP1* mutations predispose people to hypopigmented skin melanoma, uveal melanoma, mesothelioma, renal cell carcinoma, and other cancers [13]. Other potential high- to moderate-risk genes include *ACD* (also known as *TPP1*), *POT1*, and *TER2IP* coding for shelterin proteins forming a telomere-protecting complex [14]. Rare promoter mutations in telomerase (*TERT* gene) coding for an enzyme-maintaining telomere length have been found in familial melanoma [15]. An increased melanoma risk has been documented in carriers of germline mutations causing other cancer syndromes, including hereditary breast and ovarian cancer (*BRCA1/BRCA2*), retinoblastoma (*RB1*), or xeroderma pigmentosum (*XPs*) [16]. The low-risk group includes variants in genes coding for proteins involved in melanogenesis (MC1R, MITF, OCA2, SLC45A2, TYR, TYRP1) and other processes (ASIP, CASP8, MTAP, OBFC1), revealed dominantly by genome-wide association studies (GWAS) [17,18,19]. The identification of individuals carrying germline mutations in melanoma-predisposition genes enables their tailored surveillance with an early detection of melanoma and other associated tumors, and with genetic counselling for their relatives.

The Czech national cancer registry has recorded nearly doubled melanoma incidence during the past 25 years (from 7.55 cases per 100,000 inhabitants in 1994 to 13.47 in 2018), and melanoma has become the most rapidly growing malignant tumor among children and teenagers [20,21]. However, an analysis of genetic factors contributing to its development has not been performed in the Czech Republic to date.

Our study aimed primarily to characterize the spectrum and prevalence of germline mutations influencing melanoma risk. We have analyzed 264 high-risk Czech melanoma patients by panel next generation sequencing (NGS) targeting 217 genes that included eight high-to-moderate melanoma risk genes, 26 low melanoma risk genes, 37 other cancer-predisposing genes and 146 genes altered in melanoma but not associated with increased familial risk. Another task of our study was to identify melanoma patients who may benefit from genetic testing by comparing clinicopathological data from the carriers and non-carriers of germline mutations.

## 2. Materials and Methods

### 2.1. Study Population

We analyzed genomic DNA obtained from the peripheral blood of 264 unrelated melanoma patients indicated for a genetic analysis by medical geneticists based on individual or family criteria (Table 1). All patients were Caucasians of a Czech origin and provided written informed consent with the analysis approved by Ethics Committee of the General University Hospital in Prague (No.: 56/15 Grant VES 2016 AZV 1.LFUK from 2015/06/18). The patients included a subgroup of 129 individuals (97 females/32 males) indicated at the General University Hospital in Prague and 135 individuals (96 females/39 males) indicated at the Masaryk Memorial Cancer Institute in Brno. Known clinicopathological characteristics are provided in Supplementary Table S1. 

The control population included germline variants in targeted genes obtained from whole exome sequencing (WES) performed for various non-cancer conditions in 1479 unselected, adult, anonymized, ethnically matched controls (1014 males, mean age 55.5 years, range 18–88 years and 465 females, mean age 56.8 years, range 18–84). These anonymized genotypes of population-matched controls were provided by the National Center for Medical Genomics (http://ncmg.cz).

### 2.2. CZMELAC Sequence Capture Panel

The CZMELAC panel (CZech MELAnoma panel for Cancer predisposition) targeted 217 genes including (i) high-to-moderate and (ii) low melanoma risk genes, (iii) hereditary cancer syndrome genes with an uncertain melanoma risk, (iv) genes associated with “melanoma” in the Phenopedia database with at least two entries (assessed June 16, 2016; Table 2) [6,9,14,16,22,23,24,25]. 

The primary gene target for probe coverage was represented by all coding exons, including 10 bases from adjacent intronic regions, and it was designed using the NimbleDesign software (Roche) as described previously [26,27]. The final CZMELAC panel target reached 563,471 bases. Because of the strict design conditions, some repeats and homologous regions were left untargeted (Supplementary Table S2).

### 2.3. Targeted NGS Analysis

Genomic DNA was isolated from peripheral blood and 200–500 ng was used to prepare the NGS library. DNA was diluted in low TE buffer [10 mM Tris-HCl (pH 8.0) with 0.1 mM EDTA] and sheared by ultrasound (Covaris E220; Covaris, Chicago, IL, USA) to approximately 200 bp fragments checked using Agilent High Sensitivity DNA Kit on the 2100 Bioanalyzer (Agilent, Santa Clara, CA, USA). The subsequent end-repair, A-tailing, and ligation of adapters were performed using the KAPA HTP Library Preparation kit (Roche, Basel, Switzerland) according to the manufacturer with in-house prepared adapters. The processed fragments were size-selected (targeting 250–450 bp fragments) and primed with barcodes (identical to Illumina TruSeq HT index i7 and i5) by ligation-mediated PCR (LM-PCR), using in-house prepared double-indexing primers, to distinguish individual samples in subsequent pooling. The size and quality of fragments after the dual size selection and LM-PCR were controlled using Agilent High Sensitivity DNA Kit. Thirty individual samples (33 ng each) were pooled for enrichment and hybridized for 72 h with the CZMELAC panel probes (SeqCap EZ Choice Library; Roche, Basel, Switzerland). The enriched targeted sequences were amplified by post-capture PCR to create the final sequencing library. The enrichment was controlled using qPCR (NimbleGen SeqCap EZ Library SR User’s Guide). The final 15 µM library was sequenced on MiSeq using MiSeq Reagent Kit v. 3 (150 cycles; Illumina, San Diego, CA, USA) targeting 100× mean coverage per sample.

### 2.4. Bioinformatics 

The CZMELAC panel sequencing data generated in FASTQ files were analyzed as described previously [27]. Novoalign was used for mapping FASTQ files to hg19 reference. The variant-call format (VCF) files were processed by the GATK pipeline (https://software.broadinstitute.org/gatk/) from BAM files. The VCF files were annotated using SnpEff. We identified medium-size indels (insertions or duplications >35bp) using Pindel (http://gmt.genome.wustl.edu/packages/pindel/) and copy number variations (CNV) using CNVkit (https://pypi.python.org/pypi/CNVkit), using the settings that we described in detail recently [26,27]. 

### 2.5. Variant Filtration and Prioritization 

The primary list of annotated sequences was filtered in successive steps that included the elimination of: (i) low quality variants (quality < 150); (ii) out of bait variants (intergenic/deep intronic/UTR variants); (iii) intronic variants out of canonical splicing sites (±1–2 nucleotides in introns); (iv) variants with a minor allele frequency (MAF) > 0.003 in any of the ExAC/ESP6500/1000Genomes databases; (v) variants with MAF > 0.001 (*n* > 2) in 1479 population-matched controls; (vi) synonymous variants; (vii) variants referred to as benign or likely benign (B/LB) in ClinVar; (viii) variants located in the repeat masking track from the UCSC Genome Browser; (ix) frameshift/stop-gain variants in the last exon. Filtration steps ii-ix were not applied if the found variants were referred to as pathogenic/likely pathogenic (P/LP) in ClinVar or “deleterious” in our functional analyses. The dataset of the control population was filtered identically. The final set of P/LP variants included only frameshift, stop-gain, frameshifting CNV, canonical splicing, ClinVar P/LP variants, and variants classified as “deleterious” by our functional analyses. All P/LP variants (variants with very strong and strong evidence of pathogenicity according to the ACMG guidelines [28] denoted throughout this text also as “mutations”) were in melanoma patients manually inspected in IGV and, when uncertain, confirmed by Sanger sequencing. The CNV P/LP variants were confirmed by multiplex ligation-dependent probe amplification (MLPA; for *CHEK2*) or by quantitative PCR (for *SLC45A2* and *TRPM1*; Supplementary Figure S1).

### 2.6. Analysis of Splicing Alterations

All RNA samples obtained from peripheral blood or from expanded leukocytes (with/without nonsense-mediated decay inhibitor) were analyzed for splicing alterations using targeted RNA NGS with the CZMELAC panel, as described recently [29].

### 2.7. Statistical Analysis

The differences between the analyzed groups and subgroups were calculated by χ^2^ or Fischer exact tests.

### 2.8. Functional Assays for Selected Germline Variants

#### 2.8.1. CHEK2 Functional Analysis

A functional analysis of *CHEK2* VUS was performed as described recently [30]. Human RPE1-CHEK2-knock-out cells were transfected with wild-type or mutant EGFP-CHK2 and the level of KAP1-S473 phosphorylation was determined by immunofluorescence microscopy using ScanR station (Olympus, Tokyo, Japan).

#### 2.8.2. POT1 Functional Analysis 

*Cell lines and plasmids.* MCF-7 and HEK293 cells (generously provided by Rene Medema, NKI, Amsterdam) were grown in DMEM containing 6% FBS, penicillin (100 U/mL) and streptomycin (0.1 mg/mL). The cells were regularly tested for mycoplasma contamination using the MycoAlert kit (Lonza, Basel, Switzerland). A DNA fragment corresponding to human POT1 was PCR-amplified from pLPC-myc-hPOT1 (Addgene, ID:12387, Watertown, MA, USA) and inserted in frame into the XhoI/XmaI sites of pEGFP-C3. Plasmid pCDNA-3xFLAG-NLS-TPP1 was obtained from Addgene (ID: 53585, Watertown, MA, USA). Cells were transfected with plasmid DNA using polyethylenimine 40K (Polysciences, Warrington, PA, USA).

*Immunofluorescence microscopy.* To evaluate the localization of POT1 at telomeres, MCF-7 cells grown on coverslips were transfected with EGFP-POT1 or EGFP-POT1-P116L and analyzed by immunofluorescence microscopy. Cells were pre-extracted with 0.5% Triton-X 100 in ice-cold PBS for 5 min and fixed with 4% PFA for 15 min in room temperature (RT). Cells were blocked in 1% BSA for 30 min. Coverslips were incubated with TRF2 antibody (clone B-5, Santa Cruz, Dallas, TX, USA) for 2 h in RT, washed 3× in PBS, incubated with secondary antibody for 1h in RT. After washing in PBS and DAPI, coverslips were mounted with Vectashield and images were acquired using the confocal microscope Leica (Wetzlar, Germany) TCS SP8 equipped with a 63x/1.40 objective. 

*Immuno-precipitation.* The ability of POT1 to interact with the shelterin complex was evaluated by immuno-precipitation. HEK293 cells were co-transfected with FLAG-TPP1 and EGFP, EGFP-POT1 or EGFP-POT1-P116L. Cells were extracted in IP buffer (50 mM Tris pH 8.0, 120 mM NaCl, 1% Tween-20, 0.1% NP-40, 1.0% glycerol, 2 mM EDTA, 3 mM EGTA, 10 mM MgCl_2_, protease inhibitors (Roche, Basel, Switzerland) and EtBr (50 µg/mL)) and sonicated 3 × 20 sec. Clarified cell extracts were incubated with GFP-Trap beads (Chromotek, Planegg, Germany) for 1 h. After washing 4× with IP buffer, bound proteins were eluted with Laemli buffer and separated by SDS-PAGE. 

*Telomeric DNA binding assay.* POT1 binding to telomeric DNA was tested in vitro as described [31,32]. HEK293 cells transfected with EGFP, EGFP-POT1 or EGFP-POT1-P116L were extracted in IP buffer, sonicated and centrifuged for 20 min at 4 °C. Cell extracts were precleared with streptavidin sepharose beads for 1 h. Supernatants were then incubated with 2 µg of biotinylated telomeric DNA (ssG: biotin-TATATA(TTAGGG)8) or (tel5: biotin-GCAAGCTTTACCGATACAGC(TTAGGG)5) [31,32], or control DNA (ssC: biotin-TATATA(CCCTAA)8), for 12 h and Streptavidin beads were added for 1 h before washing with IP buffer. Bound proteins were eluted with Laemli buffer and analyzed by Western blotting (WB) using antibody against GFP (clone 7.1, Roche, Basel, Switzerland).

## 3. Results

### 3.1. Germline Variants in Analyzed Genes 

The overall mean coverage for all samples reached 116.7× with a good coverage uniformity across 217 analyzed genes (mean percent of target bases with coverage 20×, 50×, and 100× was 99.3%, 96.9%, and 79.2%, respectively). Panel NGS in 264 patients yielded 16,359 unique germline variants. Five hundred and sixteen of them remained after the application of variant filtration rules (described in the Methods section). Variants of uncertain significance (VUS) represented a majority (87%) of them and were excluded from further analyses as clinically inconclusive at the moment. The final 83 pathogenic/likely pathogenic (P/LP) germline variants (66 unique) in 71/264 (26.8%) melanoma patients were detected in 42/217 targeted genes (Supplementary Table S3) and included five copy number variants (CNV; two in *CHEK2* and *SLC45A2*, respectively, and one in *TRPM1*; Supplementary Figure S1). Using the identical prioritization procedure, we identified 225 P/LP variants in 204/1479 (13.8%) controls in 82/217 targeted genes, including two CNV (both in the *CHEK2* gene). Overall, 43/264 (16.3%) patients (Table 3) and 87/1479 (5.9%) controls carried a mutation in a gene previously associated with melanoma or other cancer.

#### 3.1.1. Mutations in High-to-Moderate Melanoma Risk Genes

**Figure 1:**
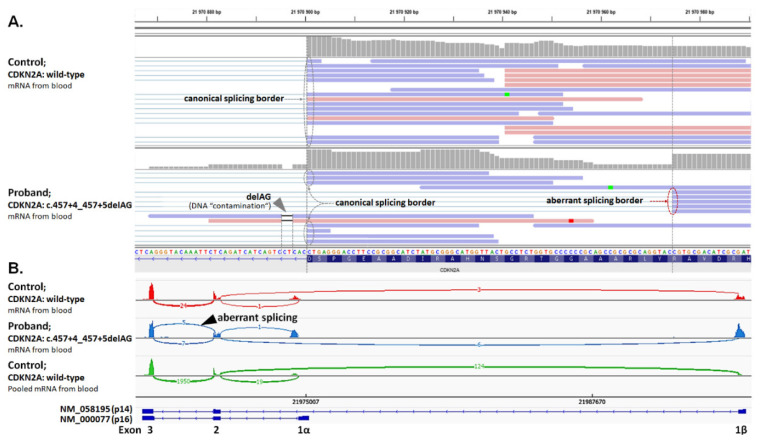
**Characterization of splicing aberrations in *CDKN2A*.** (**A**) NGS analysis of RNA isolated from blood lymphocytes identified aberrant splicing in a proband carrying the c.457+4_457+5delAG variant (visible as two reads originated from DNA “contamination”; grey arrowhead). The variant causes the elimination of the canonical splice site and activation of the cryptic splice site within exon 2, resulting in the deletion of 74 nts (r.384_457del74) and premature protein termination (p.Y129Hfs*11). (**B**) The sashimi plot shows the presence of aberrant splicing in 5/12 reads in a proband’s sample, absent in 24 reads of a control with wild-type *CDKN2A*, and another 1950 reads of 100 pooled controls.

The highest prevalence in a subgroup of high-to-moderate melanoma risk genes was found in *CDKN2A* (NM_000077). Disease-causing variants identified in six patients included ClinVar P/LP missense variants c.71G>C (p.R24P; in two patients) and c.334C>G (p.R112G), frameshift c.16_20delGGGAG (p.G6Qfs*7), in-frame c.95_112del18 (p.L32_L37del; shortening C-terminal part of ankyrin 1 domain and adjacent β-hairpin loop), and the novel splicing alteration c.457+4_457+5delAG, resulting in the activation of an aberrant splicing site (r.384_457del74) and a frameshift (p.Y129Hfs*11; Figure 1). 

**Figure 2:**
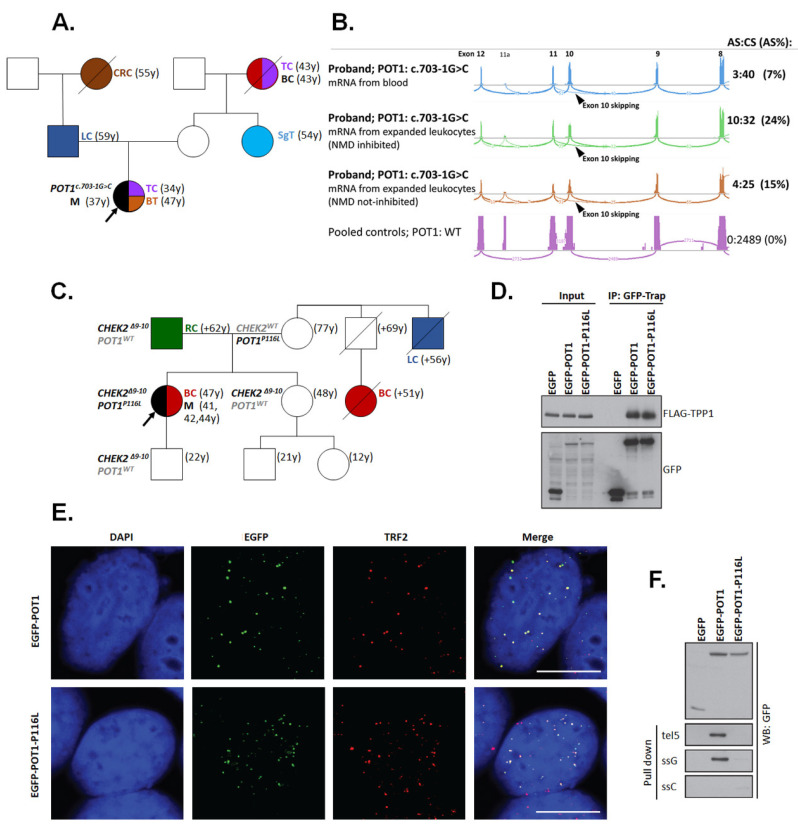
**Characterization of *POT1* germline variants.** (**A**) Family of a patient carrying c.703-1G>C. (**B**) The variant causes aberrant splicing (AS) with exon 10 skipping (r.703_869del167; arrowhead; resulting in a frameshift at the protein level: p.V235Gfs*22) that was never observed in an analysis of wild-type POT1 samples (compared in blue and purple sashimi plots). However, AS mRNA is mostly subjected to nonsense-mediated decay (NMD). The number of NGS reads of non-degraded AS products in comparison with reads from canonical splicing (CS) products increased upon the cultivation of the patient’s lymphocytes with puromycin (an NMD inhibitor; compared as green and brown plots). (**C**) Segregation of germline mutations in a family with missense p.P116L *POT1* and CNV *CHEK2* (c.909-2028_1095+330del5395) germline mutations. (**D**–**F**) Functional characterization was performed for the p.P116L *POT1* mutation. (**D**) POT1-P116L interacts with shelterin components. Extracts from cells transfected with FLAG-TPP1 (alias ACD) and EGFP, EGFP-POT1 or EGFP-POT1-P116L were immuno-precipitated using GFP-Trap. Bound proteins were analyzed with EGFP and FLAG antibodies. (**E**) POT1-P116L is able to localize to telomeres. Cells transfected with EGFP-POT1 or EGFP-POT1-P116L were fixed and stained with TRF2 antibody and analyzed using confocal microscopy. A representative image of a single plane is shown. Bar indicates 10 μm. (**F**) POT1-P116L mutant does not bind telomeric ssDNA. Extracts from cells transfected with EGFP, EGFP-POT1 or EGFP-POT1-P116L were incubated with biotinylated oligonucleotides corresponding to telomeric ssDNA (tel5 and ssG) or control DNA (ssC) and pulled down with streptavidin beads. The bound proteins were analyzed by immunoblotting using anti-GFP antibody. Abbreviations: BC—breast cancer; BT—brain tumor; CRC—colorectal cancer; LC—lung cancer; M—melanoma; RC—renal cancer; SgT—salivary gland tumor; TC—thyroid cancer.

Two germline mutations were also found in *POT1* (NM_015450). The c.703-1G>C mutation found in a proband with melanoma, dysplastic nevi, and thyroid cancer (Figure 2A) affected the canonical acceptor splice site of intron 10 resulting in exon 10 skipping at the mRNA level (r.703_869del167) and a frameshift (p.V235Gfs*22; Figure 2B). The rare missense variant c.347C>T changed the conserved amino acid p.P116L [33] in a patient with superficial spreading melanoma and breast cancer carrying also a germline deletion of 5395bp affecting exons 9 and 10 of the *CHEK2* gene (NM_007194) (Figure 2C). To dissect the functional consequences of the *POT1* missense variant inherited from the maternal branch of the family, we performed a functional analysis. First, we immuno-precipitated wild-type EGFP-POT1 or mutant EGFP-POT1-P116L from transiently transfected cells and found that both variants bound comparable levels of TPP1 (alias ACD) protein which mediates the binding of POT1 to the shelterin complex (Figure 2D). Confocal microscopy revealed that EGFP-POT1-P116L colocalized with TRF2, suggesting that it can assemble into the shelterin complex and correctly localize to telomeres (Figure 2E). Since the p.P116L mutation resides within the oligosaccharide/oligonucleotide-binding (OB1) domain [34], we hypothesized that it may impair the binding of POT1 to ssDNA. Indeed, we found that only the wild-type POT1 (but not POT1-P116L) mutant bound to the biotinylated telomeric G strand efficiently (Figure 2F). We concluded that although the p.P116L isoform can localize to telomeric dsDNA through its interaction with ACD, it fails to bind telomeric ssDNA, which makes it a functionally deleterious mutation contributing to melanoma risk.

One patient carried the c.755delA (p.D255Afs*9) mutation in *ACD* (NM_001082486), another shelterin complex gene associated with high melanoma risk [35]. This mutation results in the truncation of the POT1-binding domain of the ACD protein. Another *ACD* mutation, c.617dupT (p.H206Qfs*26), was the only P/LP variant from the category of high-to-moderate risk genes found in the control group. Although we did not find mutations in *TERT*, *BAP1*, or *CDK4*, germline mutations in the high-to-moderate risk category were present in 3.4% of patients (Table 4).

#### 3.1.2. Mutations in Low-Risk Melanoma Genes

The low-risk melanoma gene group revealed 12 carriers of mutations in 5 genes (Table 3; another *TYRP1* carrier also had a pathogenic *BRCA2* mutation). Hereditary melanoma risk was not increased in carriers of low-risk gene mutations (Table 4); however, we found a higher frequency in patients vs. controls for mutations in *TYRP1* (0.8 vs. 0%; *p* = 0.02) and *OCA2* (2.3 vs. 0.5%; OR = 4.3; 95%CI 1.2–14.2; *p* = 0.01); Supplementary Table S4.

#### 3.1.3. Mutations in Genes Associated with Hereditary Cancer Syndromes

Altogether, 22/264 (8.3%) patients (Table 3) and 57/1479 controls (3.9%) carried a P/LP variant in genes associated with hereditary cancer syndromes. Overrepresentation of mutations in patients indicated an increased melanoma risk in carriers of mutations in hereditary cancer syndrome genes (OR = 2.27; 95%CI = 1.36–3.78; *p* = 0.003); however, melanoma risk lost its significance after the exclusion of six patients carrying other concomitant mutations (Table 4). The mutations in *NBN* (OR = 10.0; 95%CI 2.5–47.0; *p* = 3.2 × 10^-4^) and *BRCA2* (OR = 9.5; 95%CI 1.8–61.4; *p* = 0.003) were the most frequent and significantly associated with hereditary melanoma. The frequencies of germline mutations in *CHEK2* gene (Supplementary Figures S1 and S2), *BRCA1*, and *MUTYH* were three times higher in patients over controls but marginally insignificant (all *p* = 0.051; Supplementary Table S4).

#### 3.1.4. Mutations in Other Genes with Unknown Familial Melanoma Risk

Mutations in 23 other genes with unknown familial melanoma risk were found in 28/264 (10.7%) patients and in a similar proportion of controls (132/1479; 8.9%). Neither the genes individually (Supplementary Table S5) nor the entire group of these genes (Table 4) were associated with a significant increase in melanoma risk.

### 3.2. Clinicopathological Characteristics of Melanoma Patients Carrying Germline Mutations

**Figure 3:**
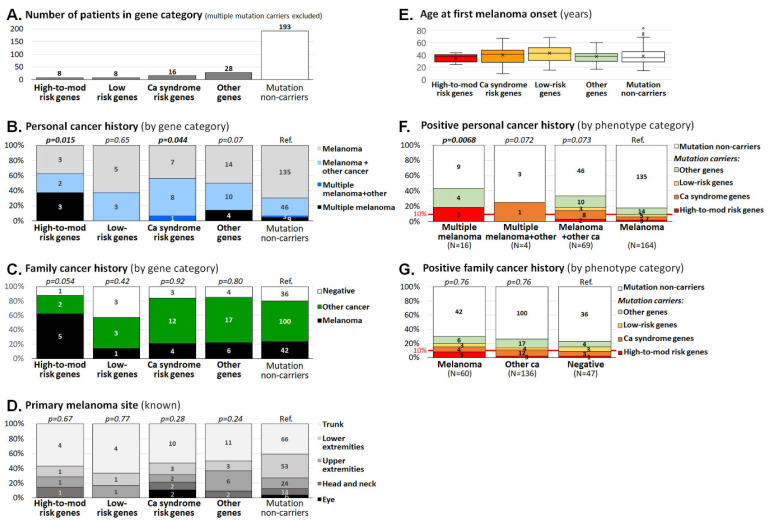
Clinicopathological characteristics of melanoma patients based on the presence of germline mutations. Panel **A** overviews the number of melanoma patients in the gene categories displayed in panels **B** to **E**. The *p*-values express significance of the differences in distribution of variables between particular category of mutation carriers and non-carriers (considered as the reference). Panel **F** and **G** display proportion of mutation carriers in analyzed gene categories in individuals with positive personal cancer history (**F**); excluding 11 multiple mutation carriers) and in individuals with known positive family cancer history (**G**); excluding 21 individuals with unknown family cancer history). Differences in proportions of carriers and non-carriers (*p*-values) in particular subgroups were calculated in patients with positive personal history (**F**) against patients with melanoma only (Ref.) and in patients with positive family cancer history (**G**) against patients with negative cancer history (Ref.).

A total of 11 carriers of more than one P/LP variant were excluded from the comparison of clinicopathological characteristics performed in the remaining 60 carriers of P/LP variants and 193 non-carriers (Figure 3A). 

Classification according to the presence of mutations in melanoma susceptibility classes (shown in Table 4) revealed an increased frequency of patients with multiple melanoma or double primary tumors among the carriers of mutations in high-to-moderate melanoma risk genes (5/8; 63% patients) and in cancer syndrome genes (9/16; 56% of patients), respectively, when compared with non-carriers (58/193; 30% of patients; Figure 3B). On the other hand, no difference was found in the presence of melanoma or other cancers in patients’ relatives, anatomical localization of melanoma, or age at melanoma onset (Figure 3C–E). The importance of personal cancer history for the potential to carry a mutation was confirmed when we calculated the proportion of patients with germline mutations in particular personal cancer history categories (Figure 3F). We noticed a significantly increased proportion of mutation carriers among patients with multiple melanoma (7/16; 44% of patients), compared with patients with single melanoma (29/164; 18% patients; *p* = 0.021). It is noteworthy that 14/89 (16%) patients with more than one tumor in personal history (i.e., patients with multiple melanoma, multiple melanoma plus other cancer, and melanoma plus other cancer) carried a mutation in a clinically relevant gene (a high-to-moderate risk melanoma gene or a cancer syndrome gene), compared with 10/164 carriers (6%) among patients with single melanoma only (*p* = 0.023). Thus, tumor multiplicity (not restricted to melanoma multiplicity) in probands increased the risk that they will carry a mutation (OR = 2.9; 95%CI 1.2–6.8). A positive family cancer history did not increase the risk of being a mutation carrier (Figure 3G); however, the prevalence of mutations in patients with a positive family cancer history (24/196 carriers, 12%) surpassed the 10% threshold justifying genetic testing in this group (in contrast to 4/47; 8.5% positively tested patients without family cancer history; *p* = 0.6).

Altogether, 7/11 double mutation carriers (excluded from the analysis of clinicopathological data) carried at least one mutation in high-risk melanoma (*POT1*/*CHEK2*) or syndromic (*ATM*/*WRN*, *BRCA1*, *BRCA2* (2x), *CHEK2*/*RAD51D*, *NBN*) genes (Table 3). Melanoma or tumor multiplicity in personal cancer history was present in four (36%) of these patients and all of them had a positive family cancer history, indicating that personal or family cancer history positivity was also more frequent among double mutation carriers.

## 4. Discussion

Our analysis demonstrated that 31/264 (11.7%) high-risk Czech melanoma patients (compared with 35/1479 or 2.3% controls) carried a mutation in some of the clinically important high-to-moderate melanoma risk genes (9 patients; 3.4%) or other cancer syndrome-associated genes (22 patients; 8.3%). As expected, *CDKN2A* was the most frequently mutated gene in the high-to-moderate risk gene group (in six analyzed patients; 2.3%). Four out of six *CDKN2A* mutation carriers developed >1 melanoma (3 patients) or other cancer (1 patient); all six carriers had a positive family cancer history and five of them had at least one relative with melanoma. The progressively rising probability of *CDKN2A* mutation prevalence with an increasing number of affected relatives with melanoma was described by Goldstein and colleagues in their study analyzing families of a European descent with at least three melanoma patients [36]. The frequency of *CDKN2A* mutation carriers rose from <40% for patients with three relatives with melanoma to >90% for those with more than six relatives with melanoma. In line with this observation, we have noticed three *CDKN2A* mutation carriers among 50 patients with one melanoma relative (6%) and two *CDKN2A* carriers among 10 patients with two melanoma relatives (20%). Goldstein et al. also observed an increased prevalence of pancreatic cancer patients in families with *CDKN2A* mutations (found in one p.R112G mutation carrier in our study). Germline mutations in high-risk melanoma susceptibility genes convey an increased risk of other cancers modifying genetic counselling in mutation carriers [24]. The spectrum of tumors in relatives diagnosed with cancer in the families of six *CDKN2A* mutation carriers included melanoma (7×), breast cancer (3×), rectal cancer (2×), and gastric, pancreatic, lung, and endometrial cancer, brain tumor, and leukemia (one each).

The three remaining patients with germline mutations in high-to-moderate melanoma risk genes carried a P/LP variant in genes coding for shelterin complex proteins. The protection of telomeres protein 1 (POT1) is essential for the control of telomere length by inhibiting telomerase [32]. In addition, POT1 prevents hyper-resection at telomeric ends by inhibiting ATR [37]. The function of POT1 at telomeres is determined by its interaction with the telomeric single-stranded 5’-TTAGGG-3’ repeats and with the TRF1/2 subunits of the shelterin complex through TPP1 (ACD) protein. Interaction with telomeric G-strand DNA is mediated by the two N-terminal OB domains of POT1, whereas the C-terminal part of POT1 interacts with TPP1 (ACD) [38]. Previous in silico and functional studies identified unstable binding and defective interaction with ssDNA for the p.R117C missense variant [33,39]. We found the adjacent p.P116L variant, described previously in a patient with sporadic cardiac sarcoma [33], in a patient with multiple melanoma and breast cancer, who also carried a large pathogenic *CHEK2* deletion. A functional analysis of the P116L isoform demonstrating its normal interaction with TPP1 (ACD) protein but impaired ssDNA binding led us to conclude that p.P116L is a functionally defective mutation. Germline *POT1* mutations have been initially described as increasing the risk of melanoma, but later studies indicate a broader cancer spectrum associated with these mutations. Notably, *POT1* mutations have recently been associated with familial non-medullary thyroid cancer [40,41,42]. A duplicity of thyroid cancer with melanoma has been identified in a patient with a newly characterized splicing *POT1* mutation (thyroid cancer was present in the patient’s untested mother’s mother). In a single melanoma patient with a negative family cancer history, we identified a mutation in the *ACD* gene truncating the C-terminal proportion of the protein containing POT1- and TINF2-interacting domains required for the localization of ACD protein into the shelterin complex. Overall, high-to-moderate risk germline mutations affecting shelterin complex genes were found in three (1.1%) analyzed patients in our study. We also detected another shelterin gene truncating mutation affecting the *TINF2* gene that we included in the low-risk gene category; however, another *TINF2* truncation has recently been described to segregate with multiple thyroid cancer and melanoma in one family [43]. A higher prevalence of mutations in *ACD*, *TERF2IP*, and *POT1* was identified in 12/132 (9.1%) high-risk *CDKN2A*/*CDK4*/*TERT*/*BAP1* wild-type European and Australian patients with multiple melanoma (≥3) [44]. A higher prevalence of germline mutations in *BAP1* (not identified in our patients) and *POT1* was also reported in a recent study by Pastorino and colleagues who identified seven carriers (2.6%) of mutations in each of these two genes among 273 Italian melanoma patients [45]. The enrollment of 22 melanoma patients with atypical Spitz nevi with relatives developing *BAP1*-related tumors can explain an increased prevalence of *BAP1* mutation carriers in this Italian study. Germline *BAP1* mutations were rarely identified in Czech patients so far, dominantly in probands with uveal melanoma or Spitz nevi [46,47].

The highest prevalence of germline mutations in our melanoma patients was found in the *NBN* gene (in 7/264 patients; 2.7%), coding for nibrin, a protein contributing to a MRN complex formation, sensing for DNA double strand breaks. We found the most frequent, Slavic founder germ-line hypomorphic variant c.657del5 in five patients [48]. Two of them also developed ovarian cancer, which was associated with *NBN* germline mutations in our population [49]. An increased melanoma prevalence among *NBN* c.657del5 mutation carriers was reported from Poland (with a frequency comparable to our patients) and southern Germany (with lower prevalence) [50,51,52]. Two of our melanoma patients (diagnosed with melanoma at 9 and 47 years, respectively; both with a melanoma-positive family cancer history) carried other rare *NBN* truncations. Gass and colleagues [53] reported a female carrier of the c.698_701del4 germline mutation developing melanoma, squamous cell carcinoma, and breast cancer with a sister suffering from melanoma and other relatives affected by various cancer types, indicating that other *NBN* truncations increase melanoma risk. Analyses of *NBN* in other cancers demonstrated a highly variable population-specific prevalence of its germline mutations. Current NCCN guidelines report an association of *NBN* mutations with an increased breast cancer risk (https://www.nccn.org/professionals/physician_gls/pdf/genetics_bop.pdf), but further studies of unselected cancer patients with carefully population-matched controls are required to determine cancer risk associated with other cancer types, including melanoma. The prevalence of *NBN* mutations but also *BRCA2* mutations was significantly (nine-fold) higher in patients than in controls. P/LP variants in *BRCA1* and *CHEK2* were less enriched in patients over controls and statistically insignificant (*p* = 0.051; Supplementary Table S5). The role of germline mutations in the breast-ovarian cancer predisposition genes *BRCA1* and *BRCA2* in the risk of familial melanoma development is still a matter of debate [54] and the exact melanoma risk increase (if any) in mutation carriers is uncertain. The same could be said of *CHEK2* as documented in a recent meta-analysis evaluating the association of germline *CHEK2* mutations with melanoma [55]. Large studies utilizing large gene panels to analyze patients with unselected melanoma or, even better, unselected cancer, will be required to dissect the risk of melanoma associated with hereditary cancer syndrome genes. However, we would like to emphasize that 4/9 *BRCA1* or *BRCA2* pathogenic mutation carriers and all *CHEK2* P/LP variant carriers would not be eligible for germline genetic testing according to the current guidelines, despite the fact that all other mutation carriers (except for one patient with the founder c.5266dupC *BRCA1* mutation) had a positive family cancer history and four also developed secondary tumors alongside solitary or multiple melanoma (Table 3). The genetic counselling was offered to all carriers of mutations in high and moderate cancer risk genes.

An analysis of clinicopathological characteristics shows not only that multiple primary melanoma patients carry an increased risk of mutations in melanoma-predisposition genes, but also that the presence of melanoma and other non-melanoma cancer in the proband increased the potential to carry a clinically meaningful mutation in a melanoma predisposition or hereditary cancer syndrome gene. 

We are aware of some limitations of our study. Most melanoma patients analyzed in our study were referred to the analysis by medical geneticists. This fact explains the enrichment of patient population in early-onset, multiple cancer, and family cancer-positive cases and incomplete clinicopathological data that lack phenotypic characteristics (eye and hair color, skin phototype according to Fitzpatrick, total number of nevi, the presence of clinically atypical nevi, freckle density, iris pigmentation), lifetime history of sunburns, and specific melanoma characteristics (histological subtype, Breslow thickness, clinical staging) in most of the patients. We are also aware that the gene selection in our CZMELAC panel would omit potentially clinically important gene(s). However, we would like to emphasize that we aimed to evaluate the importance of known melanoma/other cancer predisposition genes and candidate genes for clinical purposes in our melanoma patients rather than to identify genes that have not been associated with hereditary melanoma so far. Furthermore, only P/LP mutations were considered for subsequent statistical analyses. We excluded all VUS (except those in *CHEK2* and *POT1* that we functionally classified as deleterious) as currently clinically inconclusive, being aware that some of them may represent potentially important variants in both patient and control datasets. The presence of VUS substantially hampers the clinical utility of NGS diagnostics. Classifications of VUS frequently require demanding and time-consuming functional analyses that are beyond the expertise available in most of diagnostic laboratories. Therefore, VUS classifications, which are critically important for appropriate clinical interpretations of variants in cancer predisposition genes, are an opportunity for a collaborative effort of international consortia bringing together experts from various disciplines, who may provide substantial capacity for in vitro testing of VUS characterized by the co-operating laboratories.

In conclusion, we comprehensively assessed the prevalence of germline variants affecting currently known or candidate melanoma-predisposition genes in Czech melanoma patients and in the general population. Our analysis demonstrated that high-to-moderate risk genes, including genes coding for shelterin complex proteins, should be targeted in the multicancer panel NGS analysis. An analysis of clinicopathological characteristics indicated that patients eligible for such an analysis should not be restricted to multiple primary melanoma patients or patients with a positive familial melanoma cancer history, but they should also include melanoma patients with other primary cancer and melanoma patients with a positive family cancer history.

## Figures and Tables

**Table 1:** Characteristic of subgroups combining personal cancer history (rows) and family cancer history (FCH; columns) criteria in 264 melanoma (M.) patients enrolled in the study.

Criteria	Posit. FCH incl. M.	Posit. FCH incl. Other Cancers	Negative FCH	Unknown FCH	Patients; N (%)	Mean Age; yrs (Range)
**Multiple primary M.** **& other cancer**	0	4	0	2	6 (2.3)	45.0 (38–58)
**Multiple primary M.**	5	8	3	1	17 (6.4)	37.3 (24–75)
**M. & other cancer**	9	45	9	8	71 (26.9)	47.3 (14–83)
**M. only, dg at < 25 yrs**	5	17	11	3	36 (13.6)	20.0 (9–24)
**M. only, dg at ≥ 25 yrs**	41	62	24	7	134 (50.8)	37.1 (25–69)
**Patients; N** (% of all)	60 (22.7)	136 (51.5)	47 (17.8)	21 (8.0)	264 (100)	37.7 (9–83)
**Mean age;** yrs (range)	38.9 (9–69)	37.8 (14–83)	33.0 (15–66)	44.2 (14–75)	-	-

**Table 2:** Analyzed genes in CZEMELAC (CZech MELAnoma panel for Cancer predisposition) panel. Detailed information, including full names of analyzed genes, is provided in Supplementary Table S2.

**High-to-moderate melanoma risk genes**	*ACD, BAP1, CDK4, CDKN2A, MITF, POT1, TERF2IP, TERT*
**Low melanoma risk genes**	*AGR3, ARNT, ASIP, CASP8, CCND1, CDKN2B, CLPTM1L, FTO, HERC2, IRF4, MC1R, MGMT, MTAP, MX2, OBFC1, OCA2, PARP1, PLA2G6, SETDB1, SLC24A4, SLC45A2, TERF1, TERF2, TINF2, TYR, TYRP1*
**Hereditary cancer syndrome genes with uncertain melanoma risk**	*APC, ATM, BARD1, BMPR1A, BRCA1, BRCA2, BRIP1, CDH1, FH, CHEK2, KIT, MET, MSH2, MSH3, MSH6, NBN, NF1, NF2, PALB2, PMS2, POLD1, POLE, PTEN, RAD51C, RAD51D, RB1, RET, SDHA, SDHB, SDHC, SDHD, SMAD4, STK11, TP53, VHL, WRN, WT1*
**Genes with unknown impact on hereditary melanoma development**	*ABLIM1, APEX1, ATRN, AURKA, BBC3, BLM, BRAF, BRMS1, CASP10, CBL, CCAR2, CCNH, CDK10, CDK7, CDKN1A, CDKN1B, CDKN1C, CEBPA, COX8A, CTLA4, CTNNB1, CYP11A1, CYP17A1, CYP19A1, CYP1A1, CYP1A2, CYP3A5, DAB2IP, DCAF4, DDB1, DDB2, EDNRB, EGF, EGFR, EIF1AX, EPCAM, ERBB2, ERBB4, ERCC1, ERCC2, ERCC3, ERCC4, ERCC5, ERCC6, ERCC8, EXOC2, EZH2, FANCC, FANCL, FANCM, FAS, FASLG, FGFR2, FGFR4, FLCN, FLT1, FOXP3, GATA2, GATA4, GC, GNA11, GNAQ, GPC3, GSTM1, GSTM3, GSTP1, GSTT1, H2AFY, HRAS, IDH1, IDH2, IFIH1, IFNA1, IFNG, IL10, IL2RA, IL4, IL6, IL8, ING4, KAT6A, KIAA1967, KMT2A, KRAS, LRIG1, MAP2K1, MDM2, MLH1, MLH3, MMP1, MMP3, MUTYH, MYH7B, NCOA6, NFKB1, NFKBIE, NOD2, NOTCH3, NRAS, PAX5, PDGFRA, PIGU, PIK3CA, PIK3R1, PIK3R4, PMAIP1, PMS1, POLH, POMC, PPM1D, PPP6C, PRF1, PTGS2, PTCH1, PTPN11, PTPN22, RAC1, RAD23A, RAD23B, RASEF, RECQL, RECQL4, RHOBTB2, RUNX1, SBDS, SF3B1, SH2B3, SLX4, SMARCB1, SNX31, STAG2, STK19, SUZ12, TACC1, TERC, TLR3, TRPM1, TSC1, TSC2, VDR, XAB2, XPA, XPC, XRCC1, XRCC3, ZNF365*

**Table 3:** Germline P/LP (pathogenic/likely pathogenic) variants in melanoma patients.

^(a)^	Gene: Coding Sequence (Protein) Change - Concomitant Mutation	Mel Site (Age) ^(b)^	Other Tumors in Proband (Age)	Family Cancer History Tumor Type (N) ^(c)^
***High-to-moderate risk genes***				
**F**	*CDKN2A*: c.16_20del5 (p.G6Qfs*7)	TR (38)	none	BC (1), Leu (1), Mel (1), other 3 relatives with unknown tumors
**F**	*CDKN2A*: c.71G>C (p.R24P)	TR (24)	Mel (35)	CRC (1), Mel (1), UrC(1)
**F**	*CDKN2A*: c.71G>C (p.R24P)	TR (28)	Mel (38)	Mel (2)
**F**	*CDKN2A*: c.95_112del (p.L32_L37del)	LE (28)	GC (48)	BC (2), CRC(1), GC (1), LC (1), Mel (2)
**M**	*CDKN2A*: c.334C>G (p.R112G)	HE (43)	none	Mel (1), PaC(1)
**F**	*CDKN2A*: c.457+4_457+5delAG (p.Y129Hfs*11)	TR (29)	Mel (34)	BT (1)
**F**	*POT1*: c.347C>T (p.P116L); *- CHEK2*: c.909-2028_1095+330del5395 (p.M304Lfs*15)	UE (41)	Mel (41,42,44); BC (47)	RC (1)
**F**	*POT1*: c.703-1G>C (p.V235Gfs*22)	n.a. (37)	TC (34); BT (47)	BC (1), CRC (1), LC(1), SgT (1), TC (1)
**M**	*ACD*: c.755delA (p.D255Afs*9)	UE (39)	none	negative
***Low-risk genes***				
**F**	*OCA2*: c.1211C>T (p.T404M); *- KAT6A*: c.1138G>T (p.E380*)	n.a. (29)	none	Mel(1)
**M**	*OCA2*: c.1327G>A (p.V443I)	TR (15)	none	negative
**F**	*OCA2*: c.1327G>A (p.V443I)	TR (43)	none	BC (3), CRC (3), PaC (1)
**F**	*OCA2*: c.1327G>A (p.V443I)	LE (52)	Ly (38); SkC (49)	Leu (1), Unknown (1)
**M**	*OCA2*: c.2037G>C (p.W679C)	n.a. (50)	none	negative
**M**	*OCA2*: c.2037G>C (p.W679C)	n.a. (68)	SkC (68)	n.a.
**M**	*TYRP1*: c.1054_1057del4 (p.N353Vfs*31); *- TRPM1*: Δe2-7 (p.?)	TR (36)	none	Mel (2)
**M**	*SLC45A2*: Δe1-2 (p.?); *- GSTM3*: c.393C>A (p.Y131*)	EY (25)	none	n.a.
**F**	*SLC45A2*: Δe1-4 (p.?)	TR (42)	BC (41)	PrC (1)
**M**	*TYR*: c.650G>A (p.R217Q)	TR (37)	none	negative
**F**	*TYR*: c.1037-7T>A (p.?); *- FANCC*: c.455dupA (p.N152Kfs*9)	HE (66)	BC (52); CRC (66)	BC (2), HCC (1),
**F**	*TINF2*: c.796C>T (p.R266*)	UE (48)	none	CRC (2), GbC (1), Mel (1), PrC (2), RC (1), Sarcoma (1)
***Hereditary cancer syndrome genes***				
**F**	*NBN*: c.657_661del5 (p.K219Nfs*16)	TR (24)	none	BC (1), BT (1), Mel (1)
**F**	*NBN*: c.657_661del5 (p.K219Nfs*16)	EY (25)	none	negative
**M**	*NBN*: c.657_661del5 (p.K219Nfs*16)	TR (37)	none	n.a.
**F**	*NBN*: c.657_661del5 (p.K219Nfs*16)	HE (45)	Mel (68); OC (56)	n.a.
**F**	*NBN*: c.657_661del5 (p.K219Nfs*16)	TR (65)	OC (67)	negative
**M**	*NBN*: c.1126delG (p.D376Ifs*2)	n.a. (47)	none	LC (2), Mel (1),
**F**	*NBN*: c.1723G>T (p.E575*); *- NFKBIE*: c.165_169dup5 (p.E57Afs*51)	LE (9)	none	Mel (1)
**M**	*BRCA2*: c.475G>A (p.V159M)	UE (45)	RC (46)	HL (1)
**F**	*BRCA2*: c.1389_1390delAG (p.V464Gfs*3)	LE (47)	BC (59,59)	GC (2)
**F**	*BRCA2*: c.5682C>G (p.Y1894*)	n.a. (67)	BT (59); BC (56)	3 sisters with gynecological tumors, LC (1), retinoblastoma (1)
**M**	*BRCA2*: c.7007G>A (p.R2336H); *- IFIH1*: c.2464C>T (p.R822*)	HE (22)	none	BT (1), PrC (2), TC (1)
**M**	*BRCA2*: c.8168_8172ins4 (p.Y2726Mfs*10); *- TYRP1*: c.1254C>A (p.Y418*)	n.a. (40)	Mel (36); NHL (38)	LC (2)
**F**	*BRCA1*: c.68_69delAG (p.E23Vfs*17)	TR (47)	UrC (56); OC (57)	n.a.
**F**	*BRCA1*: c.1687C>T (p.Q563*)	EY (54)	BC (46)	OC (1)
**F**	*BRCA1*: c.4214delT (p.I1405Kfs*10); *- ATM*: c.7630-2A>C (p.?); *- MUTYH* c.1187G>A (p.G396D)	LE (46)	OC (46); BC (49)	BC (3), OC (2)
**F**	*BRCA1*: c.5266dupC (p.Q1756Pfs*74)	TR (53)	BC (54)	negative
**M**	*CHEK2*:c.909-2028_1095+330del5395 (p.M304Lfs*15)	UE (28)	none	CRC(1), Ly (1), Mel (1), MMT (1)
**M**	*CHEK2*: c.846+4_846+7del4 (p.D265-H282del)	TR (38)	none	BC (1), CRC (2)
**F**	*ATM*: c.381delA (p.V128*) *- WRN*: c.1105C>T (p.R369*)	TR (41)	Mel (50)	BC (2), PaC (1)
**F**	*ATM*: c.5932G>T (p.E1978*)	TR (35)	none	LC (1), UrC (1)
**F**	*RAD51D*: c.405+2T>C (p.?); *- CHEK2*: c.917G>C (p.G306A)	TR (26)	none	CC (1)
**F**	*RB1*: c.608-1G>T (p.?)	TR (32)	BC (45)	GbC (1), LC (1)

**^(a)^** gender: M—male; F—female. **^(b)^** Melanoma localization: EY—eye; HE—head; LE—lower extremity; TR—trunk; UE—upper extremity. **^(c)^** BC—breast cancer; BT—brain tumor; CC—cervix cancer; CRC—colorectal cancer; GC—gastric cancer; GbC—gallbladder cancer, HCC—hepatocellular cancer; (n)HL—(non)Hodgkin lymphoma; LC—lung cancer; Leu—leukemia; Ly—lymphoma; Mel—melanoma; MMT—malignant mesenchymal tumor; OC—ovarian cancer; PaC—pancreatic cancer; PrC—prostate cancer; RC—renal cancer; SgT—salivary gland tumor; SkC—skin cancer; TC—thyroid cancer; UrC—urinary cancer. The reference numbers for genes listed in this table are provided in Supplementary Table S1.

**Table 4:** Frequency of pathogenic/likely pathogenic (P/LP) germline variants in melanoma-susceptibility subgroups classified according to the risk of hereditary/familial melanoma risk. Eleven carriers of more than one P/LP variant were excluded from the analysis.

Melanoma Susceptibility Class	P/LP Variants; N (%)		OR (95%CI); *p*
	264 Patients	1479 Controls	
Multiple Mutation Carriers INCLUDED *			
High-to-moderate risk melanoma genes	9 (3.4)	1 (0.1)	52.2 (6.6–413.1); 3.2 × 10^-7^
Low-risk melanoma genes	12 (4.5)	35 (2.4)	1.9 (1.0–3.8); 0.06
Hereditary cancer syndrome genes	22 (8.3)	57 (3.9)	2.3 (1.4–3.8); 0.003
Genes with unknown familial melanoma risk	28 (10.6)	132 (8.9)	1.2 (0.8–1.8); 0.4
Multiple Mutation Carriers EXCLUDED			
High-to-moderate risk melanoma genes	8 (3.2)	1 (0.1)	48.1 (6.4–2116.9); 1.5 × 10^-6^
Low-risk melanoma genes	8 (3.2)	35 (2.4)	1.3 (0.5–3.0); 0.51
Hereditary cancer syndrome genes	16 (6.3)	57 (3.9)	1.7 (0.9–3.0); 0.09
Genes with unknown familial melanoma risk	28 (10.6)	132 (8.9)	1.2 (0.8–1.8); 0.4

* If carriers of concomitant mutations pertained to more than one risk group, they were assigned to a group with a higher risk as shown in Table 3: High-risk melanoma genes > Hereditary cancer syndrome genes > Low-risk melanoma genes > Genes with unknown familial melanoma risk.

## Supplementary Materials

The following are available online at https://www.mdpi.com/2227-9059/8/10/404/s1, Table S1: Clinicopathological characteristics of analyzed melanoma patients, Table S2: List of 217 targeted genes in CZMELAC panel, Table S3: List of 83 P/LP variants found in melanoma patients (column H) and 225 P/LP variants identified in controls, Table S4: Frequency of pathogenic/likely pathogenic germline mutations in 89 out of 217 analyzed genes identified in 264 high-risk melanoma patients or in 1479 population-matched controls, Table S5: Found germline P/LP variants in genes with unknown association to familial melanoma, Figure S1: Intragenic deletions and duplications from technical control samples with known alterations and in samples from analyzed patients. Figure S2: New *CHEK2* germline variants (p.T133A and p.Y297D) identified in two melanoma patients were functionally classified as neutral in RPE1-CHEK2-KO cell-based assay.
