# Supplementary material for: Identification of Germline Mutations in Melanoma Patients with Early Onset, Double Primary Tumors, or Family Cancer History by NGS Analysis of 217 Genes
Source: Biomedicines. 2020 Oct 9;8(10):404. doi: 10.3390/biomedicines8100404 (PMC7601281; doi:10.3390/biomedicines8100404)

**Figure S1.** **A.** **CNV found in technical control samples with known alterations (ctrl, in red) or in samples from analyzed patients (other colors).** Depression of the normalized coverage line bellow -0.6 denotes for a deletion, increase of coverage line above +0.45 (in SMARCB1) indicates a duplication. Whole gene duplication of *SMARCB1* was considered as VUS (class 3). CNV in patients’ samples were confirmed by MLPA (*CHEK2* – not shown) or qPCR (for *SLC45A2* and *TRPM1*; in B.). Differences in mean crossing point (Cp) values in bold indicate for a deletion of the analyzed exon.


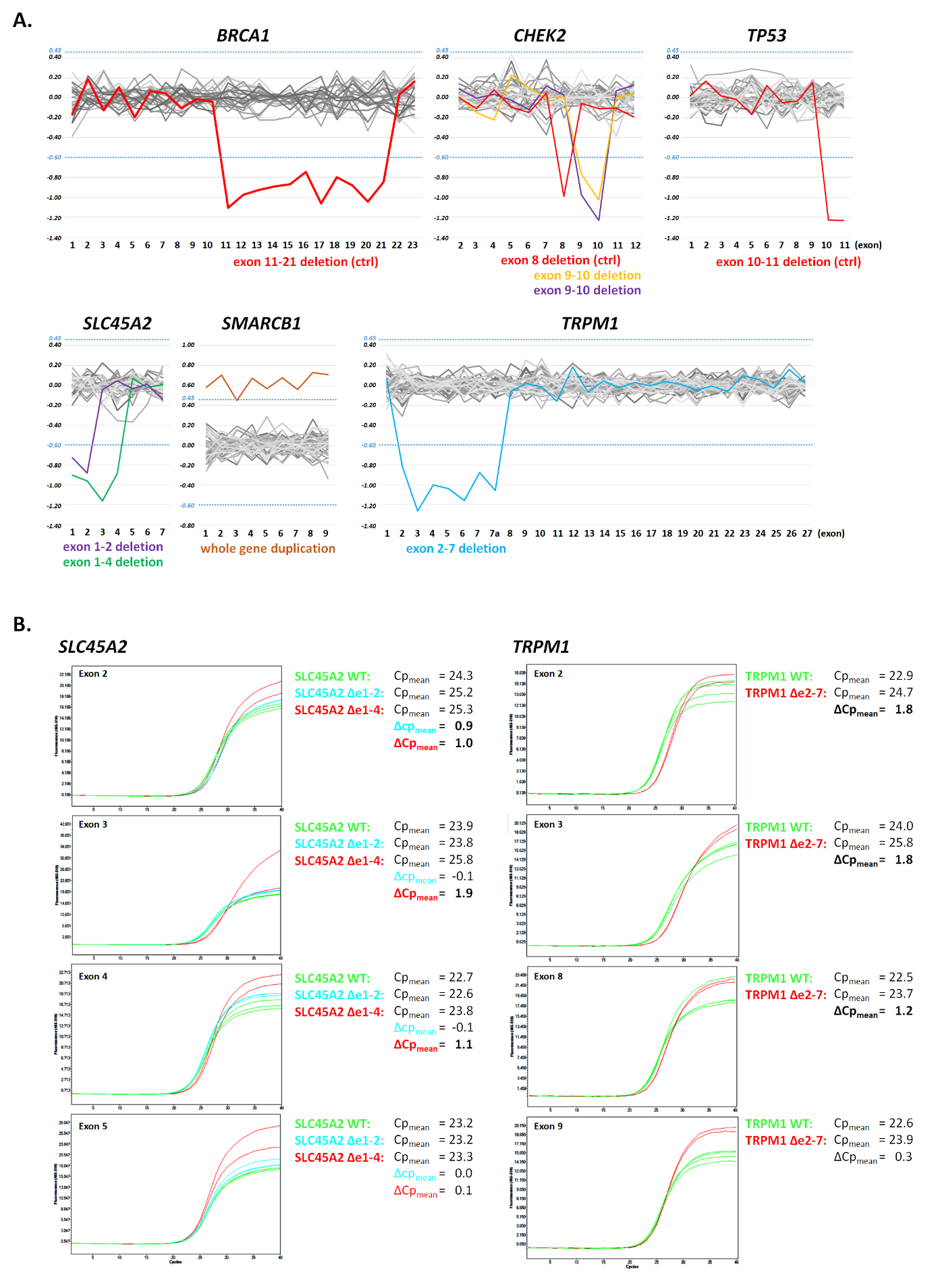


**Figure S2.** **New *CHEK2* germline variants (p.T133A and p.Y297D) identified in two melanoma patients were functionally classified as neutral in RPE1-*CHEK2*-KO cell-based assay.** The chart describes relative levels of CHK2-dependent KAP1-S473 phosphorylation in RPE1-*CHEK2*-KO cells. Variants were scored according to the CHK2 kinase activity of wild-type (WT; 100%) and truncating c.1100delC variant (0%). Variants with >50% kinase activity was scored as “neutral” (green), 25%–50% as “intermediate”, and <25% as “deleterious” (red). Error bars represent standard deviations (SD), EGFP and NT technical negative controls: RPE-CHEK2-KO cells transfected by the empty vector with eGFP (EGFP) and not transfected (NT), respectively. Other variants found in patients and controls we functionally classified previously (Kleiblova P *et al.* Int J Cancer. 2019).


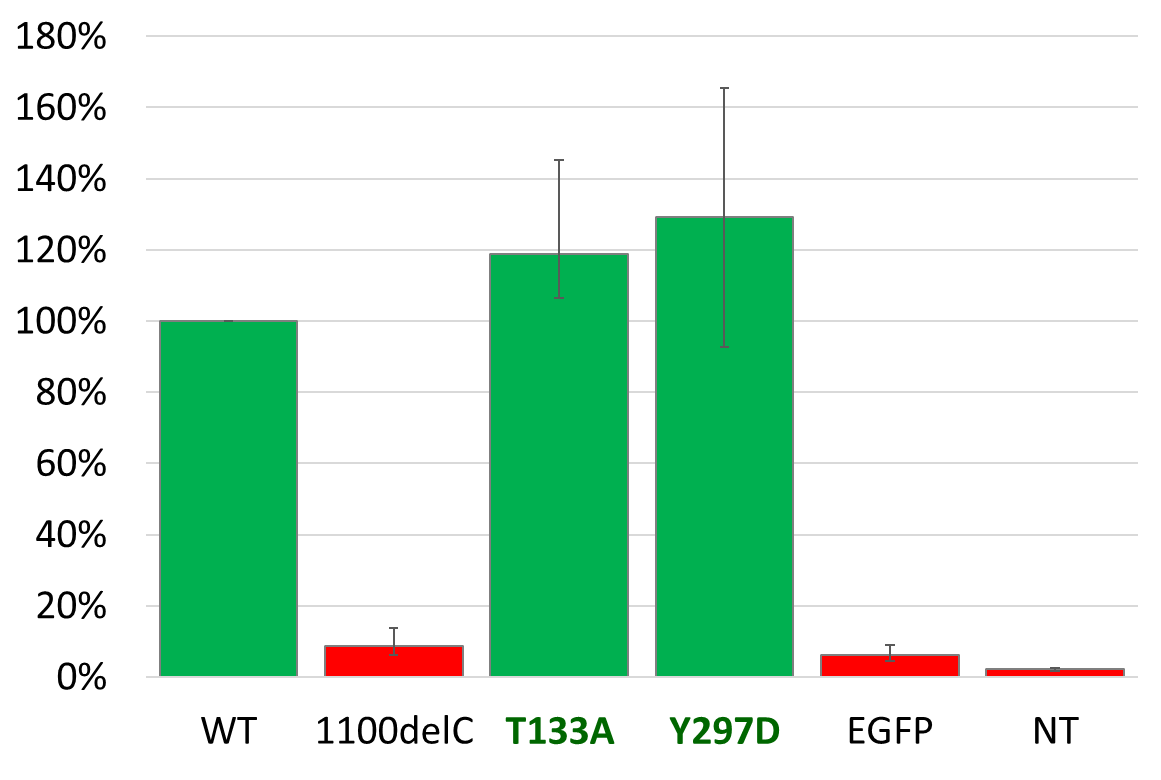

Supplement: Supplementary file 1 [file biomedicines-08-00404-s001.zip › Supplementary Figures.docx]
